# Supplementary material for: Myeloid-derived suppressor cell mitochondrial fitness governs chemotherapeutic efficacy in hematologic malignancies
Source: Nat Commun. 2024 Mar 30;15:2803. doi: 10.1038/s41467-024-47096-9 (PMC10981707; doi:10.1038/s41467-024-47096-9)
Supplement: Supplementary file 1 — Supplementary Information [file 41467_2024_47096_MOESM1_ESM.pdf]

Supplementary Information

Myeloid-derived suppressor cell mitochondrial fitness governs chemotherapeutic efficacy in hematologic malignancies

Saeed Daneshmandi [1, 2], Jee Eun Choi [1], Qi Yan [2], Cameron R. MacDonald [1], Manu Pandey [3], Mounika Goruganthu [2], Nathan Roberts [1], Prashant Singh [4], Richard M. Higashi [5], Andrew N. Lane [5], Teresa W-M. Fan [5], Jianmin Wang [6], Philip L. McCarthy [3], Elizabeth A. Repasky [1], Hemn Mohammadpour [2*]

[1]Department of Immunology, Roswell Park Comprehensive Cancer Center, Buffalo, New York, USA. [2]Department of Cell Stress Biology, Roswell Park Comprehensive Cancer Center, Buffalo, New York, USA. [3]Department of Medicine, Roswell Park Comprehensive Cancer Center, Buffalo, New York, USA. [4]Department of Cancer Genetics & Genomics, Roswell Park Comprehensive Cancer Center, Buffalo, New York, USA. [5]Department of Toxicology and Cancer Biology, Markey Cancer Center, Center for Environmental and Systems Biochemistry (CESB), Lexington, Kentucky, USA. [6]Department of Biostatistics & Bioinformatics, Roswell Park Comprehensive Cancer Center, Buffalo, New York, USA.

Correspondence: Hemn Mohammadpour (hemn.mohammadpour@roswellpark.org)

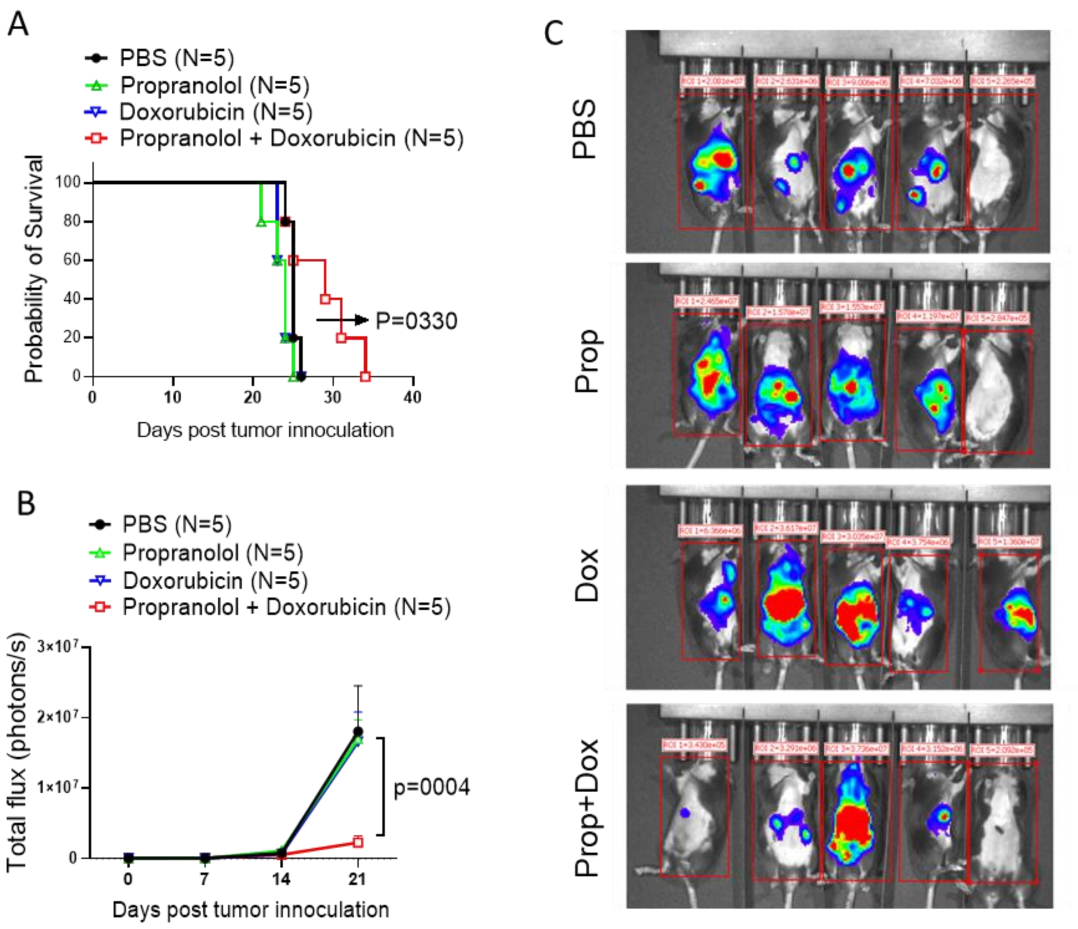

Figure S1: Combining β-adrenergic receptor (β-AR) blockade with doxorubicin results in increased survival of recipient injected with the C1498 AML cell line. (A) Survival of C1498-Luc (i.v.) mice treated with propranolol (daily; i.p.; 1 mg/kg), or doxorubicin (day 7; i.v.; 4 mg/kg), or combination of both. Two-sided Mantel-Cox log rank test. **(B-C)** Tumor growth was measured by **(B)** luciferase activity and analyzed in **(C)** Luciferase-bioluminescence image of day 21**.** Two-way ANOVA with Tukey's multiple comparison tests. Error bars represent ± SEM. Source data are provided as a Source Data file.

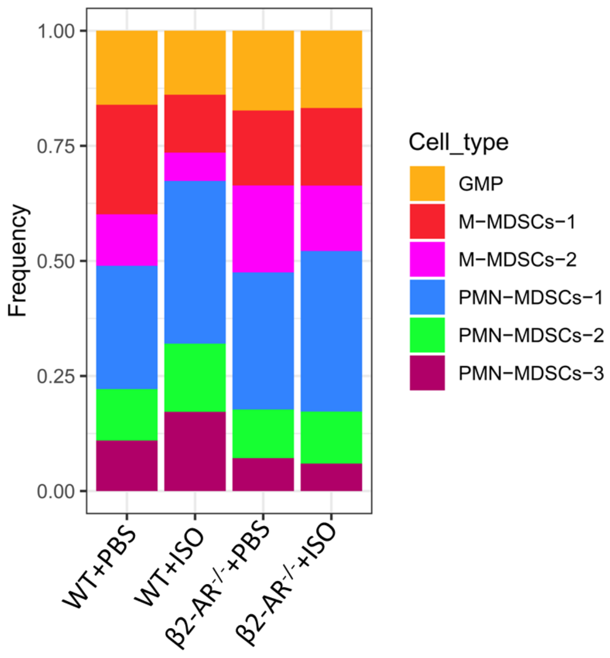

Figure S2: Frequency of MDSCs subpopulations detected by scRNA-seq. MDSCs generated *in vitro* using IL-6 (40 ng/ml) and GM-CSF (40 ng/ml) for 4 days with and without 10 µM of pan-β-AR agonist Isoproterenol (ISO) treatment from both wild type (WT) and β2AR$^{-/-}$ mice bone marrow and analyzed by single-cell RNA-sequencing (scRNA-seq). Frequency of different cell sub-populations is demonstrated. GMP: Granulocyte-monocyte progenitor; M-MDSC: Monocytic myeloid-derived suppressor cell; PMN-MDSC: Polymorphonucler myeloid-derived suppressor cell.

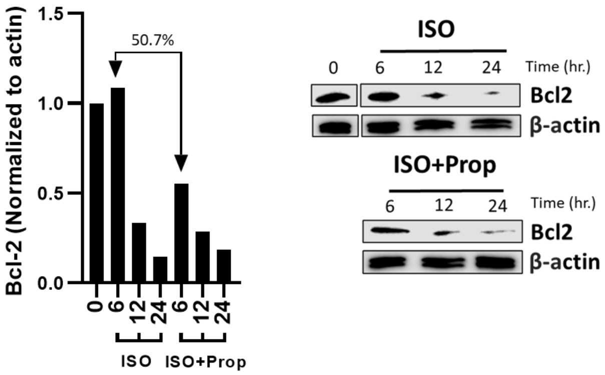

Figure S3: Western blot analysis confirming β-AR signaling induces Bcl2 expression in MDSCs. MDSCs generated *in vitro* using IL-6 (40 ng/ml) and GM-CSF (40 ng/ml) for 4 days, then treated with 10 µM of pan-β-AR agonist Isoproterenol (ISO) or ISO plus 1 µM β-AR blocker, propranolol (Prop). Cells were harvested 6 hr., 12 hr., and 24 hr. post stimulation and expression of anti-apoptotic factor Bcl2 was assessed by western blot. Source data are provided as a Source Data file.

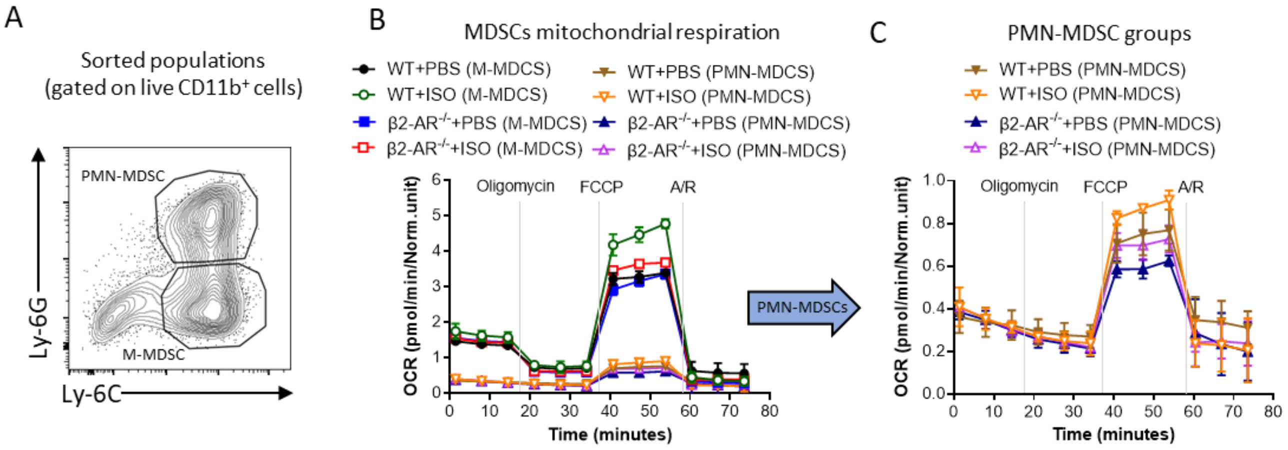

Figure S4: CD11b$^+$Ly6C$^+$Ly6G$^-$ (M-MDSC) demonstrated higher mitochondrial respiration capacity than the CD11b$^+$Ly6C$^-$Ly6G$^+$ (PMN-MDSC) subpopulation. (A-B) MDSCs were generated *in vitro* from β2AR$^{-/-}$ mice with ISO (10 µM) treatment and then sorted as **(A)** the CD11b$^+$Ly6C$^+$Ly6G$^-$ (M-MDSC) and CD11b$^+$Ly6C$^-$Ly6G$^+$ (PMN-MDSC) subpopulation. **(B)** Mitochondrial respiration was then examined using the seahorse assay in the sorted subpopulation. **(C)** Mitochondrial respiration of PMN-MDSC subpopulations from **B**. Source data are provided as a Source Data file.

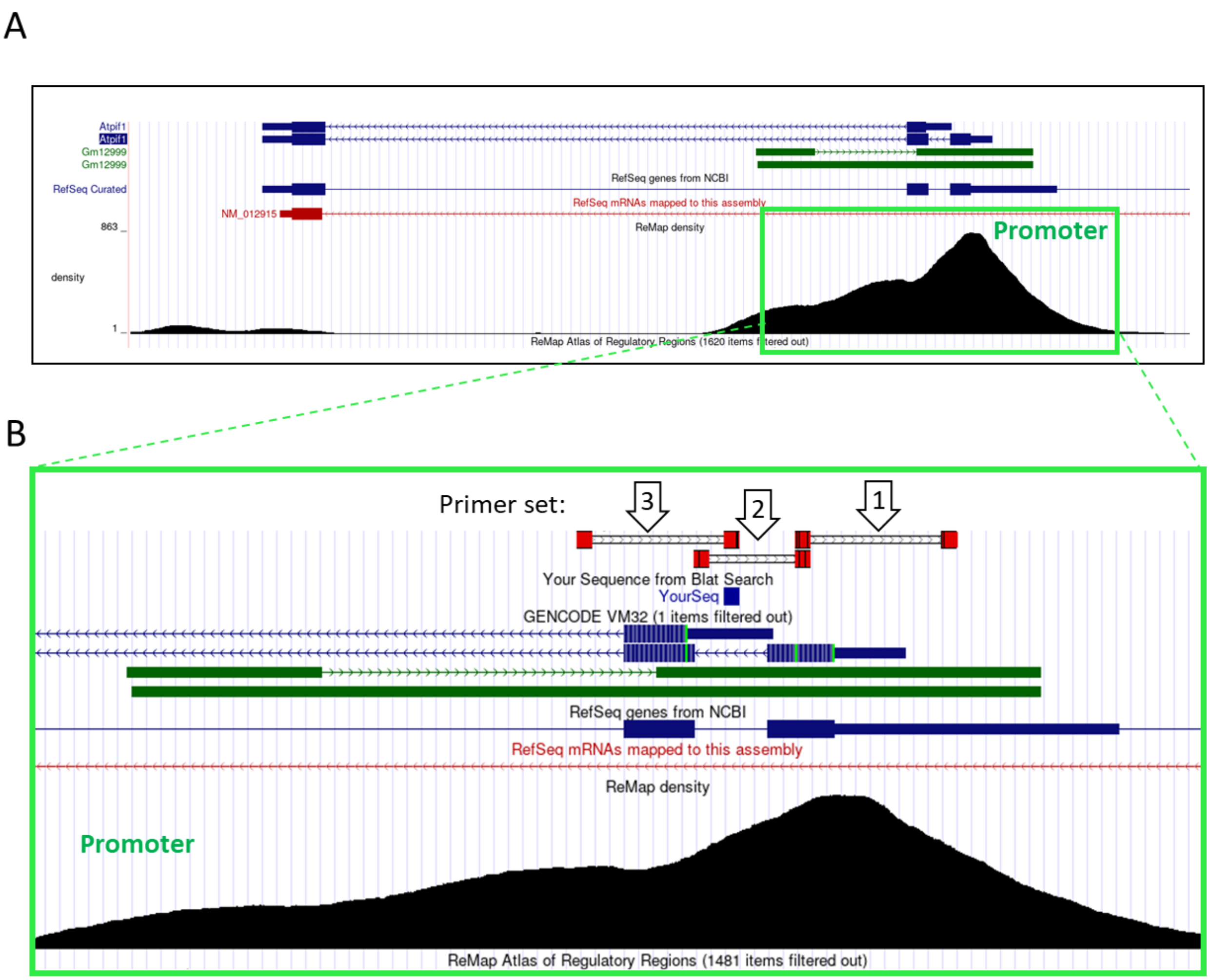

C

		Primer pairs for potential STAT3 binding sites
Site #1	Forward:	5'-ACTGCAAAGAGAGTCGTGGA-3'
	Reverse:	5'-GTTCGGTGTCTGGGGTATGA-3'
Site #2	Forward:	5'-TCATACCCCAGACACCGAAC-3'
	Reverse:	5'-AGATATCATAAGCCCCGCCC-5'
Site #3	Forward:	5'-GAGCGTAGAAAGGACAGGGA-3'
	Reverse:	5'-GCAGGTTCTCTCTCCCTTGT-3'

Figure S5: ChIP-qPCR primer sets designed for potential STAT3 binding sites in the promoter of the *Atpif1* gene.

(A-C) Potential STAT3 binding sites in the promoter of mouse *Atpif1* shown in Figure **3L**. **(A-B)** The potential pSTAT3 binding sites are demonstrated according to the UCSC Genome Browser on Mouse (GRCm39/mm39). **(C)** Primer pairs were designed for four potential STAT3 binding site confirmed by Primer 3 software and listed in the table.

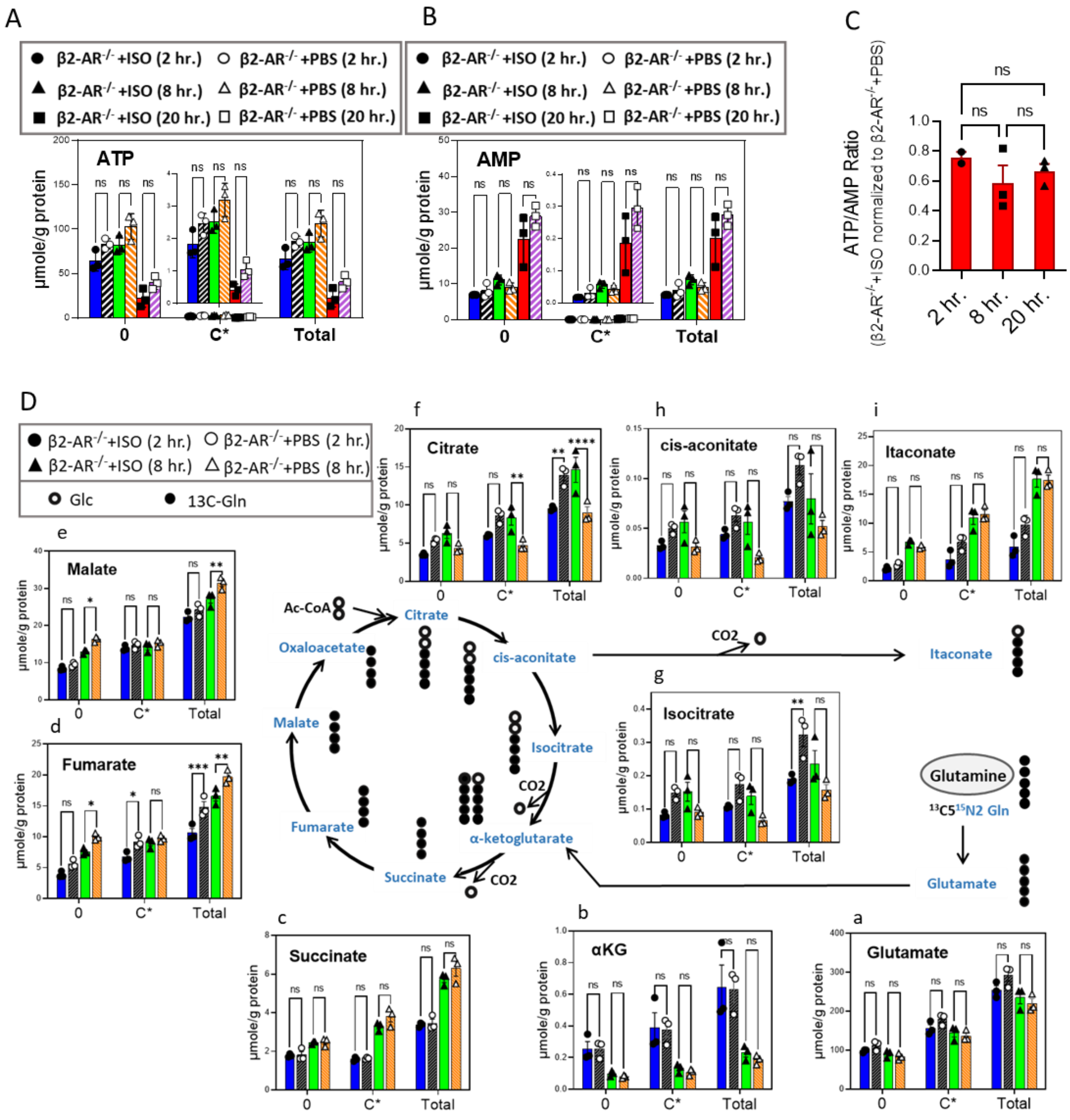

Figure S6: Isoproterenol (ISO) treatment of β2-AR$^{-/-}$ MDSCs has minimal effect on ATP/AMP generation and glutamine consumption into TCA cycle metabolites. (A-D) MDSCs were generated *in vitro* from β2-AR$^{-/-}$ mice with ISO (10 µM) treatment and then treated with $^{13}C_5$-Gln. The isotope-labeling levels in **(A)** ATP and **(B)** AMP were compared at 2 hr., 8 hr., and 20 hr. post treatment by IC-UHRMS. **(C)** The ATP/AMP ratio in β2-AR$^{-/-}$+ISO compared β2-AR$^{-/-}$+PBS control MDSCs, calculated based on the total ATP and AMP levels in the tracing experiment, indicating no significant change comparing these groups. **(D)** Glutamine transformation into TCA cycle metabolites is shown for unlabeled (0), isotope labeled (C*), and total (0+C*) metabolites. Legend in x axis: 0 = unlabeled; C* = sum of ^{13}C-labeled species with 0-x number of ^{13}C; Total = sum of all the unlabeled and isotope labeled species. Two-way ANOVA with Tukey's multiple comparison tests. (*p < 0.05; **p < 0.01; ***p <0.001). Data are presented as mean ± SEM. n = 3 per group. Error bars represent ± SEM. Source Data are provided as a Source Data file.

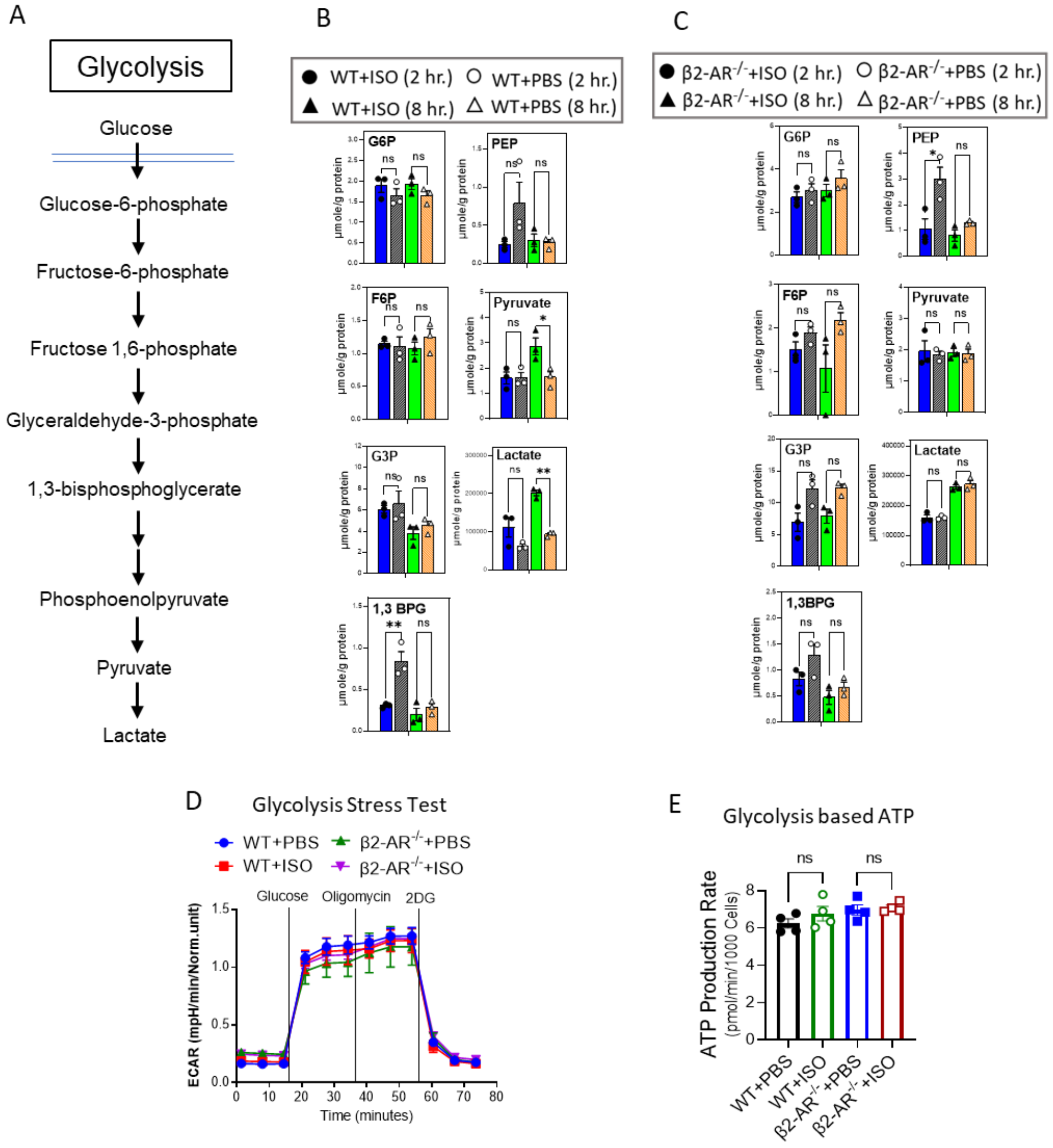

Figure S7: β-AR signaling in MDSCs does not induce a significant shift in Glycolysis pathway intermediates. MDSCs were generated *in vitro*, using IL-6 (40 ng/ml) and GM-CSF (40 ng/ml) and presence of isoproterenol (10 µM) from both WT and β2-AR$^{-/-}$ mice. **(A-B)** Generated MDSCs were treated with $^{13}C_5$-Gln as described in **Figure 5A**. **(A)** Schematic diagram demonstrating glycolysis pathway metabolites. Metabolites of glycolysis pathway are shown as a diagram. **(B-C)** Isotope-labeling amounts in glycolysis pathway metabolites were examined 2hr., and 8 hr. timepoints. In WT+PBS compared to **(B)** WT+ISO, and **(C)** β2-AR$^{-/-}$+PBS compared to β2-AR$^{-/-}$+ISO MDSCs. One-way ANOVA with Bonferroni's multiple comparison tests. (*$p < 0.05$; **$p < 0.01$). Data presented as mean ± SEM. n= 3 per group. **(D)** Agilent Seahorse XF Glycolysis Stress Test was used to examine extracellular acidification rate (ECAR) in generated MDSCs. **(E)** Agilent Seahorse XF Real-Time ATP Rate Assay was used to determine glycolysis-based ATP generation. Legend in x axis: 0 = unlabeled; C* = sum of ^{13}C-labeled species with 0-x number of ^{13}C; Total = sum of all the unlabeled and isotope labeled species. One-way ANOVA with Bonferroni's multiple comparison tests. Data presented as mean ± SEM. n= 4 per group. Error bars represent ± SEM. Source data are provided as a Source Data file.

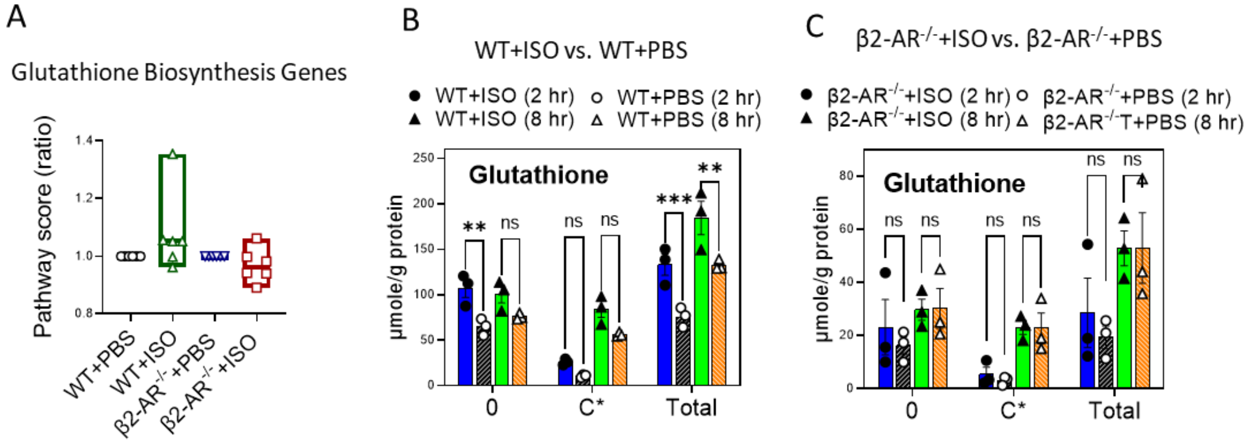

Figure S8: Glutathione biosynthesis is activated post β-AR signaling in MDSCs. (A) Pool of gene expression correlation with glutathione biosynthesis in WT+ISO compared to control MDSCs. Each data point represents pathway score value of a scRNA-seq cluster (clusters 0-5 of figure **2E-F**). **(B-C)** MDSCs were generated *in vitro* and treated with $^{13}C_5$-Gln as described in **figure 5A**. Generation of glutathione (GSH) is shown according to the isotope-labeling at 2hr., and 8 hr. timepoints for WT+ISO compared to **(B)** WT+PBS, and β2-AR$^{-/-}$+ISO compared to **(C)** β2-AR$^{-/-}$+PBS MDSCs. Legend in x axis: 0 = unlabeled; C* = sum of ^{13}C-labeled species with 0-x number of ^{13}C; Total = sum of all the unlabeled and isotope labeled species. Two-way ANOVA with Tukey's multiple comparison tests. (**p < 0.01; ***p <0.001). Data are presented as mean ± SEM. n = 3 per group. Error bars represent ± SEM. Source data are provided as a Source Data file.

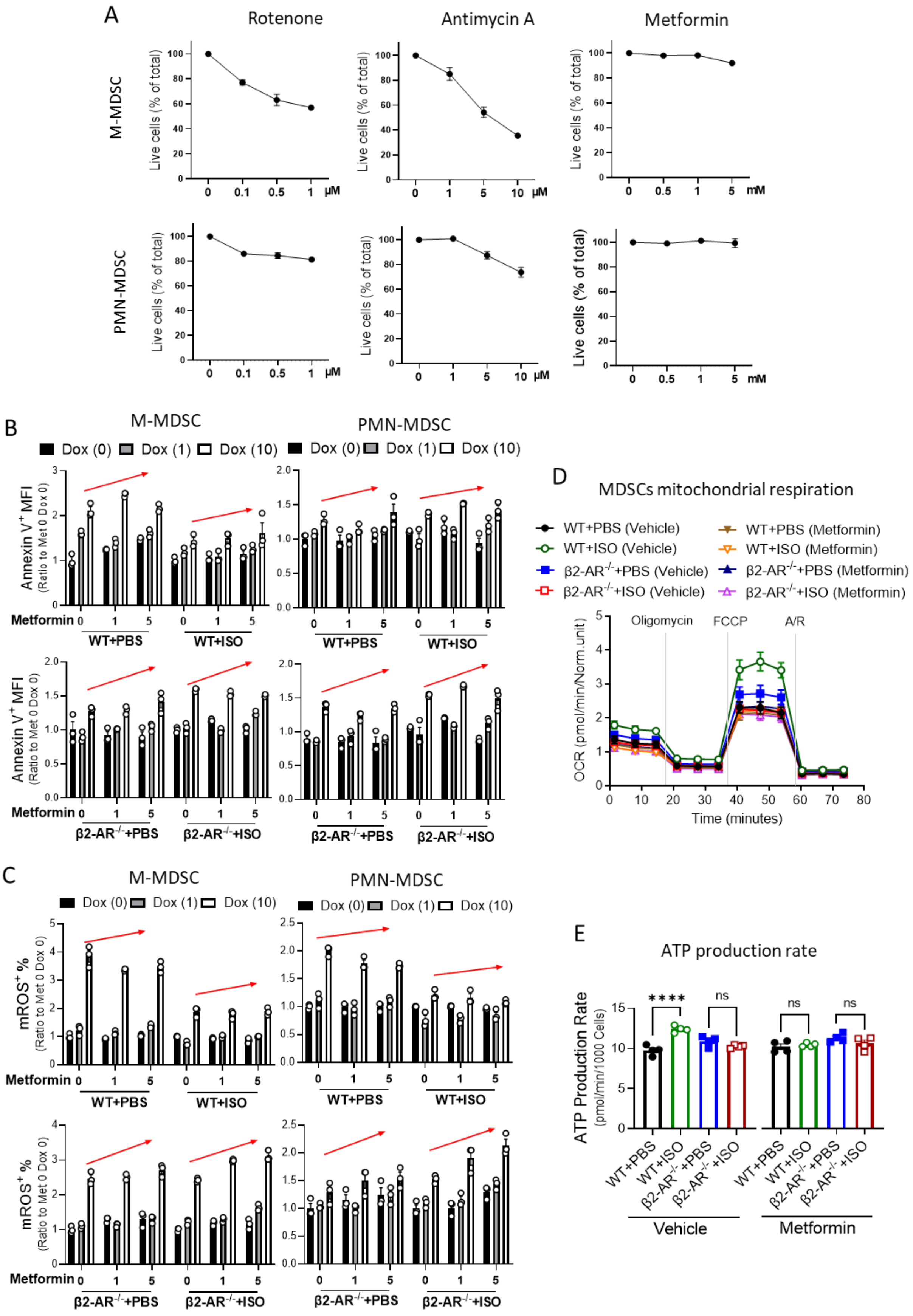

A
Rotenone
Antimycin A
Metformin
M-MDSC
PMN-MDSC
Live cells (% of total)
μM
mM
B
M-MDSC
PMN-MDSC
Dox (0) Dox (1) Dox (10)
Annexin V+ MFI (Ratio to Met 0 Dox 0)
Metformin
WT+PBS
WT+ISO
β2-AR-/-+PBS
β2-AR-/-+ISO
C
M-MDSC
PMN-MDSC
mROS+ % (Ratio to Met 0 Dox 0)
D
MDSCs mitochondrial respiration
WT+PBS (Vehicle)
WT+ISO (Vehicle)
β2-AR-/-+PBS (Vehicle)
β2-AR-/-+ISO (Vehicle)
WT+PBS (Metformin)
WT+ISO (Metformin)
β2-AR-/-+PBS (Metformin)
β2-AR-/-+ISO (Metformin)
OCR (pmol/min/Norm.unit)
Oligomycin
FCCP
A/R
Time (minutes)
E
ATP production rate
ATP Production Rate (pmol/min/1000 Cells)
****
ns
Vehicle
Metformin

Figure S9: Blocking electron transport chain (ETC) in mitochondria induces apoptosis in MDSCs. (A) MDSCs were generated *in vitro*, using IL-6 (40 ng/ml) and GM-CSF (40 ng/ml) for 4 days and treated with increasing concentrations of Rotenone (0-1 µM), Antimycin A (0-10 µM), and Metformin (0-5 mM). After 20 hrs. of treatment, viability was measured by Annexin V staining (live cell: $Aqua^-$ $Annexin V^-$) on $CD11b^+Ly6C^+Ly6G^-$ (M-MDSC), and $CD11b^+Ly6C^-Ly6G^+$ (PMN-MDSC). **(B-C)** MDSCs were generated *in vitro*, in presence of isoproterenol (10 µM) from wild type (WT) and $\beta 2AR^{-/-}$ mice and treated with increasing concentrations Metformin (0-5 mM) and doxorubicin (0-10 ng/ml). **(B)** Apoptosis rate was measured by Annexin V staining on M-MDSCs and PMN-MDSCs. **(C)** mROS production was measured by MitoSox Red staining on M-MDSCs and PMN-MDSCs. **(D-E)** MDSCs were generated as **(A)** and **(D)** mitochondrial respiration and **(E)** ATP production was evaluated by seahorse assay. One-way ANOVA with Bonferroni's multiple comparison tests. (****p <0.0001). Data presented as mean ± SEM. n= 4 per group. Error bars represent ± SEM. Source data are provided as a Source Data file.

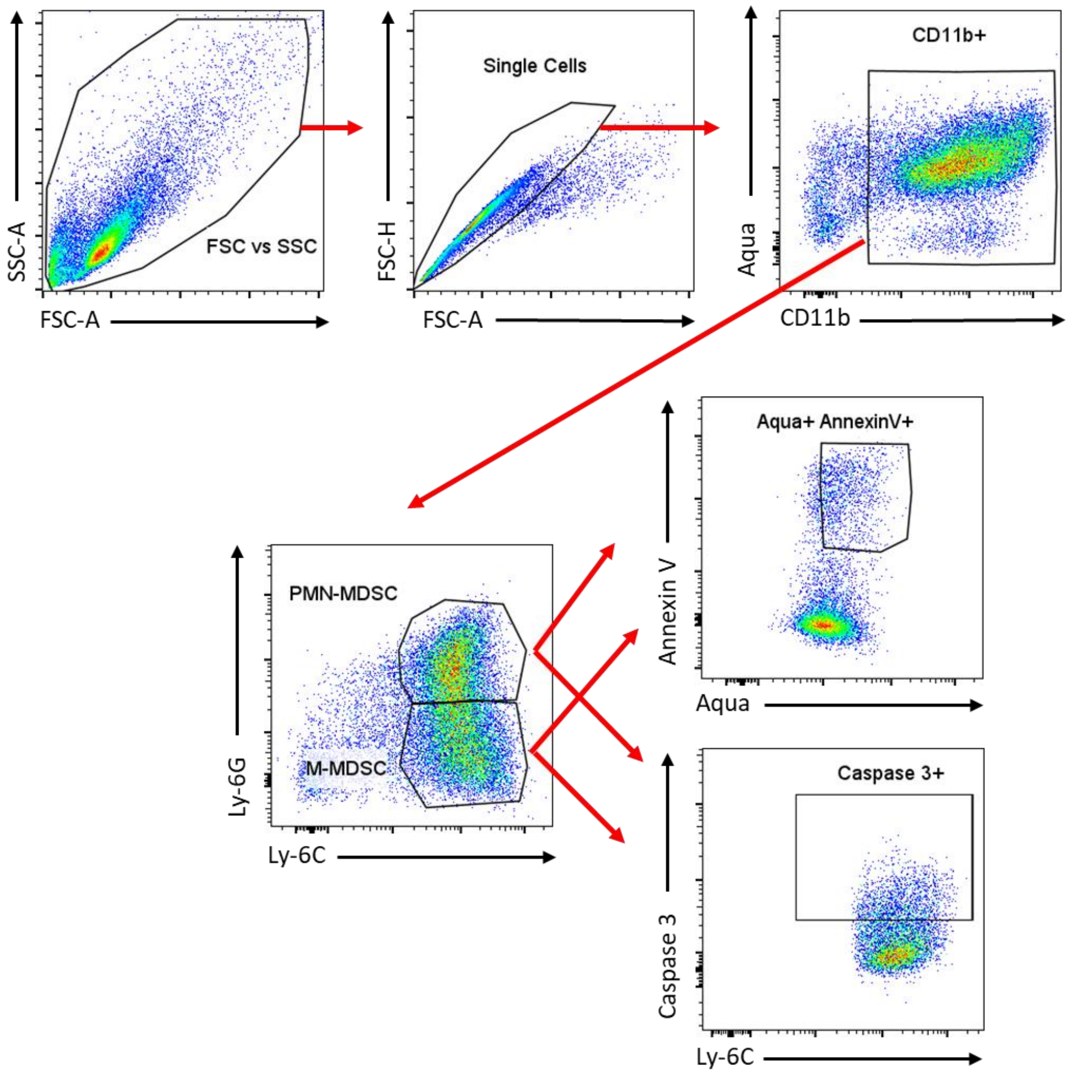

Figure S10: FACS sequential gating strategy to examine MDSC populations. (A) Gating strategy to examine the apoptosis markers on the CD11b$^+$Ly6C$^+$Ly6G$^-$ cells (M-MDSC) and CD11b$^+$Ly6C$^+$Ly6G$^+$ cells (PMN-MDSC). The same gating strategy was used to examine other functional markers I M-MDSCs and PMN-MDSCs.
