# Supplementary material for: Myeloid-derived suppressor cell mitochondrial fitness governs chemotherapeutic efficacy in hematologic malignancies
Source: Nat Commun. 2024 Mar 30;15:2803. doi: 10.1038/s41467-024-47096-9 (PMC10981707; doi:10.1038/s41467-024-47096-9)
Supplement: Supplementary file 3 — Reporting Summary [file 41467_2024_47096_MOESM3_ESM.pdf]

nature portfolio

Corresponding author(s): Hemn Mohammadpour

Last updated by author(s): Feb 1, 2024

Reporting Summary

Nature Portfolio wishes to improve the reproducibility of the work that we publish. This form provides structure for consistency and transparency in reporting. For further information on Nature Portfolio policies, see our Editorial Policies and the Editorial Policy Checklist.

Statistics

For all statistical analyses, confirm that the following items are present in the figure legend, table legend, main text, or Methods section.

n/a	Confirmed	
☐	☒	The exact sample size (*n*) for each experimental group/condition, given as a discrete number and unit of measurement
☐	☒	A statement on whether measurements were taken from distinct samples or whether the same sample was measured repeatedly
☐	☒	The statistical test(s) used AND whether they are one- or two-sided<br>*Only common tests should be described solely by name; describe more complex techniques in the Methods section.*
☐	☒	A description of all covariates tested
☐	☒	A description of any assumptions or corrections, such as tests of normality and adjustment for multiple comparisons
☐	☒	A full description of the statistical parameters including central tendency (e.g. means) or other basic estimates (e.g. regression coefficient) AND variation (e.g. standard deviation) or associated estimates of uncertainty (e.g. confidence intervals)
☐	☒	For null hypothesis testing, the test statistic (e.g. *F*, *t*, *r*) with confidence intervals, effect sizes, degrees of freedom and *P* value noted<br>*Give P values as exact values whenever suitable.*
☒	☐	For Bayesian analysis, information on the choice of priors and Markov chain Monte Carlo settings
☒	☐	For hierarchical and complex designs, identification of the appropriate level for tests and full reporting of outcomes
☒	☐	Estimates of effect sizes (e.g. Cohen's *d*, Pearson's *r*), indicating how they were calculated

Our web collection on statistics for biologists contains articles on many of the points above.

Software and code

Policy information about availability of computer code

Data collection	TraceFinder_V5 software to collect tracing experiment data
Data analysis	TraceFinder_V5 software to analyze tracing experiment data; FlowJo_V10 software to analyze flow cytometery data; Graphpad Prism_V9 to perform statistical analysis on different data set.

For manuscripts utilizing custom algorithms or software that are central to the research but not yet described in published literature, software must be made available to editors and reviewers. We strongly encourage code deposition in a community repository (e.g. GitHub). See the Nature Portfolio guidelines for submitting code & software for further information.

Data

Policy information about availability of data

All manuscripts must include a data availability statement. This statement should provide the following information, where applicable:

- Accession codes, unique identifiers, or web links for publicly available datasets
- A description of any restrictions on data availability
- For clinical datasets or third party data, please ensure that the statement adheres to our policy

The raw and processed sequencing data (single cell RNA-seq) generated in this study have been deposited in the Gene Expression Omnibus (GEO) database under accession number GSE254512. All data supporting the findings of this study are available within the article or in the Supplementary Information. Source data are provided with this paper.

Human research participants

Policy information about studies involving human research participants and Sex and Gender in Research.

Reporting on sex and gender	This study does not include human subjects.
Population characteristics	This study does not include human subjects.
Recruitment	This study does not include human subjects.
Ethics oversight	This study does not include human subjects.

Note that full information on the approval of the study protocol must also be provided in the manuscript.

Field-specific reporting

Please select the one below that is the best fit for your research. If you are not sure, read the appropriate sections before making your selection.

- [x] Life sciences
- [ ] Behavioural & social sciences
- [ ] Ecological, evolutionary & environmental sciences

For a reference copy of the document with all sections, see nature.com/documents/nr-reporting-summary-flat.pdf

Life sciences study design

All studies must disclose on these points even when the disclosure is negative.

Sample size	The sample size in different experimental groups were determined based on previous studies or preliminary studies.
Data exclusions	Data were not excluded.
Replication	Experiments were done in more that triplicate and each experiment was repeated for two or more repeats to confirm reproducibility.
Randomization	Mice in in vivo experiments were randomly distributed in different groups before treatments.
Blinding	The readout for some of the key experiments including in vivo treatments and outcome measurements were done by a third investigator (blinding). The repeats for some of the key in vitro experiments also were done by a third investigator (blinding).

Reporting for specific materials, systems and methods

We require information from authors about some types of materials, experimental systems and methods used in many studies. Here, indicate whether each material, system or method listed is relevant to your study. If you are not sure if a list item applies to your research, read the appropriate section before selecting a response.

Materials & experimental systems

n/a	Involved in the study	
[ ]	[x]	Antibodies
[ ]	[x]	Eukaryotic cell lines
[x]	[ ]	Palaeontology and archaeology
[ ]	[x]	Animals and other organisms
[x]	[ ]	Clinical data
[x]	[ ]	Dual use research of concern

Methods

n/a	Involved in the study	
[x]	[ ]	ChIP-seq
[ ]	[x]	Flow cytometry
[x]	[ ]	MRI-based neuroimaging

Antibodies

Antibodies used

Anti-mouse CD11b Antibody (clone: M1/70) BUV395 conjugated BD Biosciences Cat No#563553; RRID: AB_2738276
Anti-mouse Ly-6C Antibody (clone: HK1.4) Brilliant Violet 421™ conjugated BioLegend Cat No#128032; RRID: AB_2562178
Anti-mouse Ly-6G Antibody (clone: 1A8) APC conjugated BioLegend Cat No#127614; RRID: AB_2227348
Anti-mouse Ly-6G Antibody (clone: 1A8) PE conjugated BD Biosciences Cat No#551461; RRID: AB_394208
Anti-mouse Nos2 (iNOS) Antibody (clone: W16030C) PE conjugated BioLegend Cat No#696806; RRID: AB_2876745
Anti-human/mouse Arginase 1/ARG1 Antibody (Polyclonal) FITC conjugated R&D Systems Cat No#IC5868F; RRID: AB_10718118
Anti-mouse/human ATPIF1 Antibody (Clone: 5E2D7) Alexa Fluor® 488 conjugated Abcam Cat No#ab198075; RRID: AB_10861497

	Anti-mouse/human ATPIF1 Antibody (Clone: 5E2D7) Unconjugated ThermoFisher Cat No#A-21355; RRID: AB_2535841<br>Anti-mouse NRF2 Antibody (clone: D1Z9C) PE conjugated Cell Signaling Cat No#14409; RRID: AB_2798474<br>Purified anti-NRF2 Antibody (clone: W19086B) Unconjugated BioLegend Cat No#939201; RRID: AB_2892502<br>AMPKα Rabbit mAb (clone: D5A2) Unconjugated Cell Signaling Cat No# 5831 RRID: AB_10622186<br>Anti-AMPK-alpha, phospho (Thr172) (clone: 40H9) Unconjugated Cell Signaling Cat No# 2535 RRID: RRID:AB_331250<br>Vinculin (clone: E1E9V) Unconjugated Cell Signaling Cat No# 13901 RRID: AB_2728768<br>Anti-rabbit IgG antibody (polyclonal) Horseradish peroxidase–conjugated Cell Signaling Cat No# 7074; RRID: AB_2099233<br>Anti-mouse IgG antibody (polyclonal) Horseradish peroxidase–conjugated Cell Signaling Cat No# 7076; RRID: AB_330924
Validation	Anti-mouse CD11b Antibody (clone: M1/70); Reactivity and QC at: RRID: AB_2738276<br>Anti-mouse Ly-6C Antibody (clone: HK1.4); Reactivity and QC at: RRID: AB_2562178<br>Anti-mouse Ly-6G Antibody (clone: 1A8); Reactivity and QC at: RRID: AB_2227348<br>Anti-mouse Ly-6G Antibody (clone: 1A8); Reactivity and QC at: RRID: AB_394208<br>Anti-mouse Nos2 (iNOS) Antibody (clone: W16030C); Reactivity and QC at: RRID: AB_2876745<br>Anti-human/mouse Arginase 1/ARG1 Antibody (Polyclonal); Reactivity and QC at: RRID: AB_10718118<br>Anti-mouse/human ATPIF1 Antibody (Clone: 5E2D7); Reactivity and QC at: RRID: AB_10861497<br>Anti-mouse/human ATPIF1 Antibody (Clone: 5E2D7); Reactivity and QC at: RRID: AB_2535841<br>Anti-mouse NRF2 Antibody (clone: D1Z9C); Reactivity and QC at: RRID: AB_2798474<br>Purified anti-NRF2 Antibody (clone: W19086B); Reactivity and QC at: RRID: AB_2892502<br>AMPKα Rabbit mAb (clone: D5A2); Reactivity and QC at: RRID: AB_10622186<br>Anti-AMPK-alpha, phospho (Thr172) (clone: 40H9); Reactivity and QC at: RRID: RRID:AB_331250<br>Vinculin (clone: E1E9V); Reactivity and QC at: RRID: AB_2728768<br>Anti-rabbit IgG antibody (polyclonal) Horseradish peroxidase–conjugated; Reactivity and QC at: RRID: AB_2099233<br>Anti-mouse IgG antibody (polyclonal) Horseradish peroxidase–conjugated; Reactivity and QC at: RRID: AB_330924

Eukaryotic cell lines

Policy information about cell lines and Sex and Gender in Research

Cell line source(s)	EL4 cell line; ATCC; ATCC® TIB-39. EL4 is a T lymphoblast that was established from a lymphoma induced in a C57BL mouse by 9,10-dimethyl-1,2-benzanthracene. This cell line can be used in immunology research.
Authentication	The EL4 cell line was purchased from ATCC and the authentication was done by ATCC.
Mycoplasma contamination	The cell line was tested negative for Mycoplasma contamination
Commonly misidentified lines<br>(See ICLAC register)	There was no misidentified cell line in this study.

Animals and other research organisms

Policy information about studies involving animals; ARRIVE guidelines recommended for reporting animal research, and Sex and Gender in Research

Laboratory animals	C57BL/6J (B6 CD45.2+) from The Jackson Laboratory with Stock No: 000664. Sex: Female; Age: 6-10 weeks old.<br>C57BL/6NJ from The Jackson Laboratory with Stock No: 005304. Sex: Female; Age: 6-10 weeks old.<br>C57BL/6NJ-Acod1em1(IMPC)J/J from The Jackson Laboratory with Stock No: 029340. Sex: Female; Age: 6-10 weeks old.<br>B6.129P2-Lyz2tm1(cre)Ifo/J (LysMCre) from The Jackson Laboratory with Stock No: : 004781. Sex: Female; Age: 6-10 weeks old.<br>C57BL/6J (β2-AR-/-) Sex: Female; Age: 6-10 weeks old.
Wild animals	This study did not involve wild animals.
Reporting on sex	The sex was reported for each experiments. Experiments were done on female mice.
Field-collected samples	This study did not involve field-collected samples.
Ethics oversight	Animal work was done in accordance with the Institutional Animal Care and Use Committee (IACUC)-approved protocol according to Roswell Park animal care guidelines (protocol #1143M).

Note that full information on the approval of the study protocol must also be provided in the manuscript.

Flow Cytometry

Plots

Confirm that:

- [x] The axis labels state the marker and fluorochrome used (e.g. CD4-FITC).
- [x] The axis scales are clearly visible. Include numbers along axes only for bottom left plot of group (a 'group' is an analysis of identical markers).
- [x] All plots are contour plots with outliers or pseudocolor plots.
- [x] A numerical value for number of cells or percentage (with statistics) is provided.

Methodology

Sample preparation	To obtain cells from the spleen or bone marrow of mice, tissues were harvested and mechanically disintegrated, passed through a 70 µm strainer, followed by red blood cell (RBC) lysis. For in vitro cultured cells, MDSCs were harvested 4 days post differentiation.
Instrument	Cells were analyzed using the BD LSRFortessa™ Cell Analyzer (BD Bioscience).
Software	Cells were analyzed using FlowJo V10 software.
Cell population abundance	The purity of in vitro generated MDSCs were more that 90% (CD11b+).
Gating strategy	M-MDSC: FSC/SSC, Single cell on FSC/FSH, SSC on Aqua- for Live cell, then Aqua-CD11b+, then Ly6C+Ly6G-<br>PMN-MDSC: FSC/SSC, Single cell on FSC/FSH, SSC on Aqua- for Live cell, then Aqua-CD11b+, then Ly6C-Ly6G+<br>M-MDSC (Caspase 3+): FSC/SSC, Single cell on FSC/FSH, SSC on Aqua- for Live cell, then Aqua-CD11b+, then Ly6C+Ly6G-Caspase3+<br>PMN-MDSC (Caspase 3+): FSC/SSC, Single cell on FSC/FSH, SSC on Aqua- for Live cell, then Aqua-CD11b+, then Ly6C-Ly6G +Caspase 3+<br>M-MDSC (Nrf2+): FSC/SSC, Single cell on FSC/FSH, SSC on Aqua- for Live cell, then Aqua-CD11b+, then Ly6C+Ly6G-Nef2+<br>PMN-MDSC (Nrf2+): FSC/SSC, Single cell on FSC/FSH, SSC on Aqua- for Live cell, then Aqua-CD11b+, then Ly6C-Ly6G+Nrf2+<br>M-MDSC (Annexin V+): FSC/SSC, Single cell on FSC/FSH, SSC/CD11b+, Ly6C+Ly6G-, then Aqua+Annexin V+<br>PMN-MDSC (Annexin V+): FSC/SSC, Single cell on FSC/FSH, SSC/CD11b+, Ly6C-Ly6G+, then Aqua+Annexin V+<br>M-MDSC (mROS+): FSC/SSC, Single cell on FSC/FSH, SSC on Aqua- for Live cell, then Aqua-CD11b+, then Ly6C+Ly6G-mROS+<br>PMN-MDSC (mROS+): FSC/SSC, Single cell on FSC/FSH, SSC on Aqua- for Live cell, then Aqua-CD11b+, then Ly6C-Ly6G+mROS+

- [x] Tick this box to confirm that a figure exemplifying the gating strategy is provided in the Supplementary Information.
